# New Three-Finger Protein from Starfish *Asteria rubens* Shares Structure and Pharmacology with Human Brain Neuromodulator Lynx2

**DOI:** 10.3390/md20080503

**Published:** 2022-08-03

**Authors:** Alexander S. Paramonov, Mikhail A. Shulepko, Alexey M. Makhonin, Maxim L. Bychkov, Dmitrii S. Kulbatskii, Andrey M. Chernikov, Mikhail Yu. Myshkin, Sergey V. Shabelnikov, Zakhar O. Shenkarev, Mikhail P. Kirpichnikov, Ekaterina N. Lyukmanova

**Affiliations:** 1 Shemyakin-Ovchinnikov Institute of Bioorganic Chemistry, Russian Academy of Sciences, Miklukho-Maklaya Str. 16/10, 119997 Moscow, Russia; apar@nmr.ru (A.S.P.); mikhailshulepko@gmail.com (M.A.S.); ammakhonin@edu.hse.ru (A.M.M.); maksim.bychkov@gmail.com (M.L.B.); d.kulbatskiy@gmail.com (D.S.K.); chernikov.andrei.m@gmail.com (A.M.C.); mikhail.myshkin@phystech.edu (M.Y.M.); zakhar-shenkarev@yandex.ru (Z.O.S.); kirpichnikov@inbox.ru (M.P.K.); 2 AI Centre, National Research University Higher School of Economics, Myasnitskaya Str. 20, 101000 Moscow, Russia; 3 Interdisciplinary Scientific and Educational School of Moscow University “Molecular Technologies of the Living Systems and Synthetic Biology”, Faculty of Biology, Lomonosov Moscow State University, Leninskie Gory, 119234 Moscow, Russia; 4 Institute of Cytology, Russian Academy of Sciences, Tikhoretsky Prospect 4, 194064 St. Petersburg, Russia; buddasvami@gmail.com; 5 Moscow Institute of Physics and Technology, State University, Institutskiy Per. 9, 141701 Moscow, Russia

**Keywords:** Ly6/uPAR, three-finger proteins, LU-domain, nAChR, Lynx2, Lypd6, Lynx1, Lypd6b

## Abstract

Three-finger proteins (TFPs) are small proteins with characteristic three-finger β-structural fold stabilized by the system of conserved disulfide bonds. These proteins have been found in organisms from different taxonomic groups and perform various important regulatory functions or act as components of snake venoms. Recently, four TFPs (Lystars 1–4) with unknown function were identified in the coelomic fluid proteome of starfish *A. rubens*. Here we analyzed the genomes of *A. rubens* and *A. planci* starfishes and predicted additional five and six proteins containing three-finger domains, respectively. One of them, named Lystar5, is expressed in *A. rubens* coelomocytes and has sequence homology to the human brain neuromodulator Lynx2. The three-finger structure of Lystar5 close to the structure of Lynx2 was confirmed by NMR. Similar to Lynx2, Lystar5 negatively modulated α4β2 nicotinic acetylcholine receptors (nAChRs) expressed in *X. laevis* oocytes. Incubation with Lystar5 decreased the expression of acetylcholine esterase and α4 and α7 nAChR subunits in the hippocampal neurons. In summary, for the first time we reported modulator of the cholinergic system in starfish.

## 1. Introduction

Three-finger proteins (TFPs), also known as Ly6/uPAR proteins, are found in a wide range of different organisms, including those that are phylogenetically distant. The most famous and well-studied representatives of TFPs are the components of snake venom. In addition, TFPs have been found in mammals, birds, amphibians, fish, and arthropods [1]. 

A common feature of TFPs is a conserved fold represented by so called LU-domain [2], which includes three elongated loops (or “fingers”) and a compact β-structural core ("head"), which is stabilized by four invariant disulfide bonds. In addition to these disulfide bonds, many members of the Ly6/uPAR protein family have one to three additional disulfide bonds in the “fingers”. Some TFPs are tethered to a cell membrane via the GPI anchor, while others represent the secreted molecules [1,3]. 

Most of the mammalian TFPs studied to date (Lynx1, Lynx2 (LYPD1), Lypd6, Lypd6b, SLURP-1, SLURP-2) belong to the nervous system or to the epithelium [4,5,6,7,8,9,10,11,12] and target different receptors, for example, nicotinic acetylcholine receptors (nAChRs). In contrast to snake toxins, which are typically high-affinity receptor inhibitors, mammalian TFPs do not exhibit complete inhibition of the receptors, but modulate them over the micromolar concentration range [7,8,12,13,14,15,16,17]. Other TFPs regulate the complement system in the mammal blood (CD59 [3]), are a part of plasminogen activation system (uPAR is urokinase receptor [18]), or even modulate viral infection (Ly6E [19,20,21]).

TFPs in other organisms also play many essential functions. Thus, Lypd6 of fish and frog participate in the embryogenesis regulation [22]. Li16 of frog *Rana sylvatica* is involved in the mechanism of adaptation to the winter hibernation [23]. TFP of zebrafish *Danio rerio* has been shown to act as an antimicrobial peptide [24]. In salamander, TFPs participate in limb regeneration processes [25] or can serve as regulators of sexual behavior [26]. In insects, TFPs interact with the Nlα1/β2-nAChR receptors, affecting sensitivity to insecticides [27] or may be responsible for sleep by interacting with nAChRs and Shaker K^+^ channel [28,29].

Recently, four TFPs (Lystar1-4) were identified in the coelomic fluid of *Asterias rubens* by the proteomic analysis [30], but the exact role and structural features of these proteins remain unknown. Here, we performed bioinformatic search for TFPs in genomes of starfishes *Asteria rubens* [31,32,33] and *Acanthaster planci* [34,35,36] and found five and six additional proteins containing LU-domains, respectively. One protein from *A. rubens* demonstrated a high degree of sequence homology with the human neuromodulator Lynx2 (LYPD1) from the brain [9,37]. This protein, called by us as Lystar5, is encoded by the three-exon gene (Gene ID: 117291096), and its expression on mRNA level in *A. rubens* coelomic cells was confirmed by real-time PCR. Then we developed a bacterial expression system for recombinant production of Lystar5 and confirmed its three-finger spatial structure by NMR. Electrophysiological studies of Lystar5 in *Xenopus* oocytes revealed the inhibition of α4β2-nAChRs—the target of Lynx2, which inhibits activation of this receptor and influences its desensitization and ACh sensitivity [9,38]. Incubation of hippocampal neurons with Lystar5 resulted in down-regulation of the expression of α4 and α7 nAChR subunits as well as of acetylcholine esterase. Data obtained indicate the ancient origin of the Ly6/uPAR proteins and emphasize their essential role for living organisms from echinoderms to higher animals.

## 2. Results

### 2.1. Identification of TFPs in Starfish Genomes

**Figure 1:**
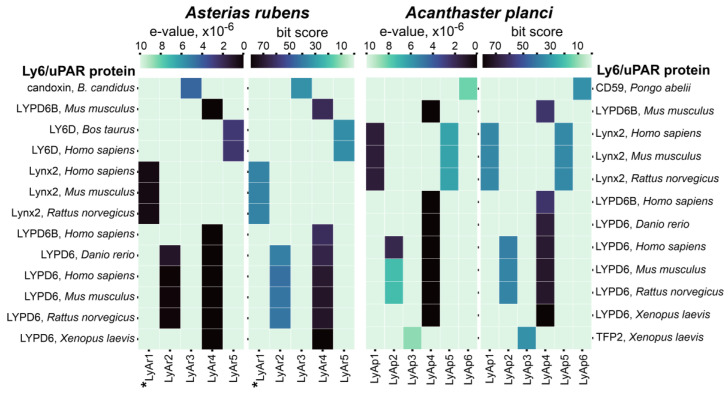
E-value and bit score heatmaps for putative TFPs from *A. rubens* and *A. planci* predicted by BLAST search vs. closest Ly6/uPAR proteins. Darker cells correspond to higher bit-scores and lower e-values of blast hit. The sequence of LyAr1 from *A. rubens* studied in this work (Lystar5) is designated by asterisk. Correspondence of putative TFPs from starfishes to the transcript IDs in the NCBI database is given in Table S2.

To search TFPs in starfish, we compiled a list of 153 known TFPs from various organisms containing a single LU-domain. This list included TFPs from mammals, amphibians, insects, birds, fishes, flatworms, and viruses, as well as snake three-finger toxins (Table S1). The genome of non-toxic starfish *A. rubens* [31,32,33] was analyzed. In addition, to find possible three-finger toxins, we analyzed the genome of toxic starfish *A. planci* [34,35,36], which feeds on polyps of reef-building corals and affects the population of endangered coral reefs [39,40]. BLAST search with restrictions on e-value (<10^−5^) resulted in five and six possible TFPs in *A. rubens* and *A. planci*, respectively. For easy reading, we named these sequences as LyAr1-5 for *A. rubens* and LyAp1-6 *A. planci* (Table S2). These putative proteins demonstrated a sequence homology with frog TFP2, snake toxin candoxin, and the proteins CD59, LY6D, Lynx2, LYPD6, and LYPD6B having human, rat, mice, bovine, fish, and frog origin (Figure 1).

**Figure 2:**
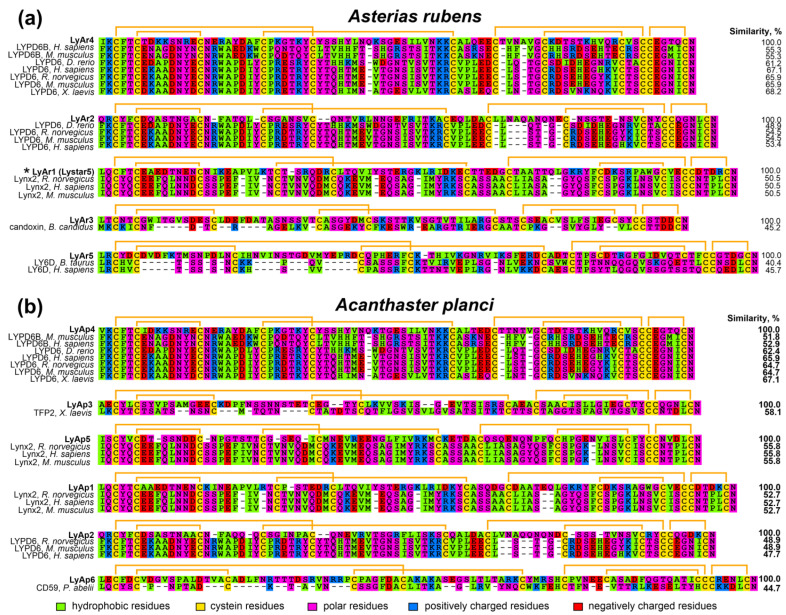
Alignment of amino acid sequences of BLAST hits from *A. rubens* (**a**) and *A. planci* (**b**) with corresponding TFPs. Disulfide bonds are shown by orange brackets. The sequence of LyAr1 from *A. rubens* studied in this work (Lystar5) is designated by asterisk. Correspondence of putative TFPs from starfishes to the transcript IDs in the NCBI database is given in Table S2 (supplementary file).

Analysis of the amino acid sequences of the BLAST hits revealed that the highest homology (close to 50% or higher) was observed between *A. rubens* LyAr4/LyAr6 and mammalian LYPD6/LYPD6B proteins; *A. rubens* LyAr1 and mammalian Lynx2; *A. planci* LyAp4 and mammalian LYPD6/LYPD6B; *A. planci* LyAp3 and frog TFP2; *A. planci* LyAp1/LyAp5 and mammalian LYPD1; and *A. planci* LyAp2 and mammalian LYPD6 (Figure 2). Other found putative TFPs (*A. rubens* LyAr3 and LyAr5, and *A. planci* LyAp6) demonstrated homology with known TFPs less than 50%. This, given that the content of conserved Cys residues is ~15–16% of the LU-domain amino acid sequence, makes the homology with snake toxin candoxin, mammalian LY6D, and orangutan CD59 rather speculative. Nevertheless, these starfish proteins, if expressed, will also have the three-finger structure. 

### 2.2. Relationships of TFPs from Starfishes and Other Animals

**Figure 3:**
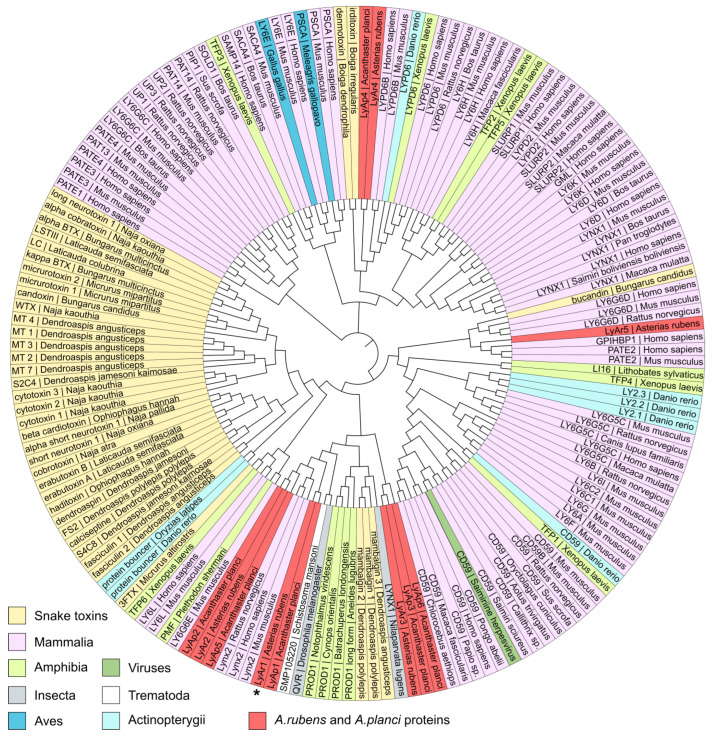
Analysis of TFPs clusters from different taxonomic groups including proteins from starfishes *A. rubens* and *A. planci*. The sequence of LyAr1 from *A. rubens* studied in this work (Lystar5) is designated by an asterisk. Correspondence of putative TFPs from starfishes to the transcript IDs in the NCBI database is given in Table S2.

To predict the possible function of putative starfish TFPs and their relationships with the proteins from other organisms, we used the guide tree (Figure 3) obtained from the multiple sequence alignment (MSA) of the extracted LU-domains of TFPs from the different taxonomic groups (Table S1, supplementary file) together with the protein sequences found in *A. rubens* and *A. planci* (Figure 1). As a result, the putative *A. planci* proteins LyAp2, LyAp5, and LyAp1, and *A. rubens* sequences LyAr1 and LyAr2 were grouped together with mammalian neuromodulator Lynx2. However, the NCBI automatic annotator predicts that LyAp2 and LyAr2 are parts of larger sequences belonging to adhesion G-protein coupling receptors. Therefore, their grouping with Lynx2 is irrelevant. Indeed, the BLAST analysis revealed larger similarity of these sequences to LYPD6 (48–55%) rather than to Lynx2. In contrast, the other putative starfish proteins from this group demonstrated better alignment with mammalian Lynx2 (similarity 50–56%, Figure 1 and Figure 2, Table S3). 

The second group included starfish proteins LyAr4 (*A. rubens*) and LyAp4 (*A. planci*), and regulators of the Wnt-signaling LYPD6/LYPD6B from different organisms (Figure 3). This grouping is in agreement with the results of the BLAST analysis (similarity 52–67%, Figure 1 and Figure 2, Table S4). The putative protein LyAr5 (*A. rubens*) was grouped with human endothelial protein GPIHBP1 and mammalian protein from the reproductive system PATE2. Similar to the case described above, this grouping is not supported by the BLAST analysis, indicating similarity less than 45%. Finally, the LyAp3 and LyAp6 sequences from *A. planci* and LyAr3 from *A. rubens* formed the isolated group (Figure 3, Table S5).

Summarizing the obtained results, from the nine putative TFPs found, the three sequences (LyAr1, LyAp1, and LyAp5) are similar to mammalian Lynx2 and two proteins (LyAr4 and LyAp4) are similar to LYPD6/LYPD6B. 

**Figure 4:**
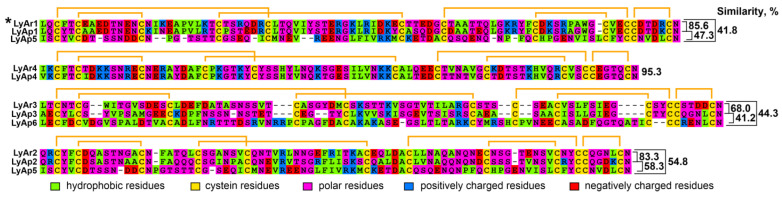
Alignment of amino acid sequences of TFPs found in *A. rubens* and *A. planci*. Disulfide bonds are shown by orange brackets. The sequence of LyAr1 from *A. rubens* studied in this work (Lystar5) is designated by an asterisk.

Analysis of the amino acid sequence similarity of the determined putative proteins (Figure 4) revealed the presence of highly similar pairs of sequences in *A. rubens* and *A. planci*. The LyAr4/LyAp4, LyAr2/LyAp2 (the fragments of GPCRs), LyAr1/LyAp1, and LyAr3/LyAp3 pairs demonstrate the internal similarity >68% and most likely represent the orthologs. At the same time, the sequences LyAp5 and LyAp6 (*A. planci*), and LyAr5 (*A. rubens*) most likely represent unique proteins.

### 2.3. Protein LyAr1 (Lystar5) Is Expressed in Asteria rubens

In order to confirm the LyAr1 (XP_033628517.1) mRNA expression in *A. rubens* (the protein is coded by the three-exon Gene XM_033772626.1), we extracted total RNA and synthesized corresponding cDNA from coelomocytes of the starfish. Real-time PCR was performed with two sets of primers: the first one amplified a cDNA fragment from the promoter zone to the exon 2 beginning, while the second one amplified the cDNA fragment from the end of exon 2 to the beginning of exon 3. Both primer pairs were separated by introns to confirm the absence of amplification of products from genomic DNA. Analysis of the PCR products by gel electrophoresis revealed the presence of two oligonucleotides with length of ~180 and ~200 b.p. (Figure S1). The products lengths and melting temperatures (T_m_) corresponded to the theoretical values (184 b.p. and Tm ~ 80 °C for the first primer pair and 200 b.p. and T_m_ ~ 90 °C for the second one). This confirmed the LyAr1 mRNA expression in *A. rubens* coelomocytes (Figure S1). The corresponding protein was named Lystar5.

### 2.4. Recombinant Production and Purification of Lystar5

To study a function and structure of Lystar5, we developed a recombinant expression system for production of this protein in *E. coli*. Analysis of the LyAr1 amino acid sequence predicted the presence of a signal peptide at the *N*-terminus and a GPI-anchor at the *C*-terminus of Lystar5 (Figure S2). In our work we used the gene encoding the LU-domain of Lystar5 without the flanking *N*- and *C*-terminal sequences. Previously we developed an approach for direct cytoplasmic expression of TFPs in the form of inclusion bodies with subsequent renaturation [37,41,42]. Here we used this protocol with slight changes in the composition of renaturation buffer (see Methods section). The final yield of refolded Lystar5 and its ^13^C-^15^N-labeled analog was ~0.1 mg per 1 liter of bacterial culture. The homogeneity and purity of refolded Lystar5 was confirmed by analytical HPLC and mass spectrometry (Figure S3). The measured molecular mass (10,307 Da) coincided with the theoretical mass of Lystar5 molecule with an additional *N*-terminal Met residue and six closed disulfide bonds.

### 2.5. NMR Study Confirms the Three-Finger Fold of Lystar5

NMR study of the Lystar5 spatial structure was carried out using the ^13^C,^15^N-labeled protein. Due to low protein yield, we obtained the protein sample with a concentration of only about 40 μM. This allowed us to measure a number of triple resonance spectra in which the signals of the most spin systems were observed. However, this concentration was insufficient to obtain NOESY spectra with an acceptable signal-to-noise ratio. We were able to obtain a ^15^N-NOESY-HSQC spectrum with a mixing time of 200 ms, but even in this case, many cross-peaks had an intensity comparable to the noise level. Therefore, it was impossible to measure a sufficient number of proton-proton distances and the calculation of the spatial structure was not carried out. 

**Figure 5:**
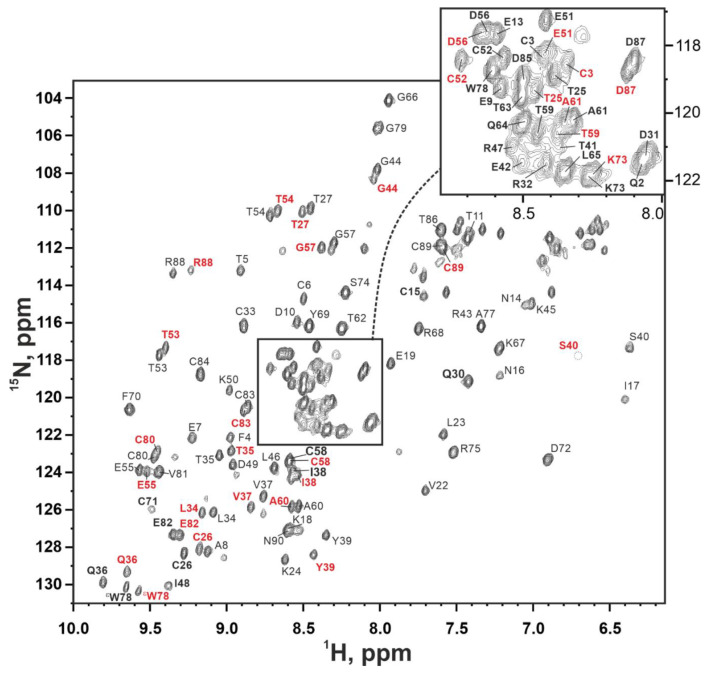
^15^N-HSQC NMR spectrum of ^13^C,^15^N-labelled Lystar5 (37 °C, pH 7.0, 800 MHz). Cross-peaks of the form I labeled in black, cross-peaks of the form II are labeled in red where they differ.

The almost complete assignment of backbone resonances (H^N^, N, H^α^, C, C^α^) was obtained (Figure 5, Table S6). Five of the 88 expected signals of amide groups were not observed in the spectra, most likely due to the signal broadening induced by fast solvent exchange in the vicinity of charged groups. 

The two signals of amide groups were observed for some residues in the ^15^N-HSQC spectrum (Figure 5 and Figure S4a). This signal duplication indicated the presence of two structural forms of the protein (form I and form II). Since the sample was homogeneous according to the mass-spectra and SDS-PAGE (Figure S2), we can conclude that these structural forms are the result of the conformational exchange process going slow on the NMR timescale. Measurement of the ^15^N-HSQC signal intensities in the temperature range from 15 to 45 °C revealed temperature dependent interconversion of the two forms of the protein (Figure S3b).

**Figure 6:**
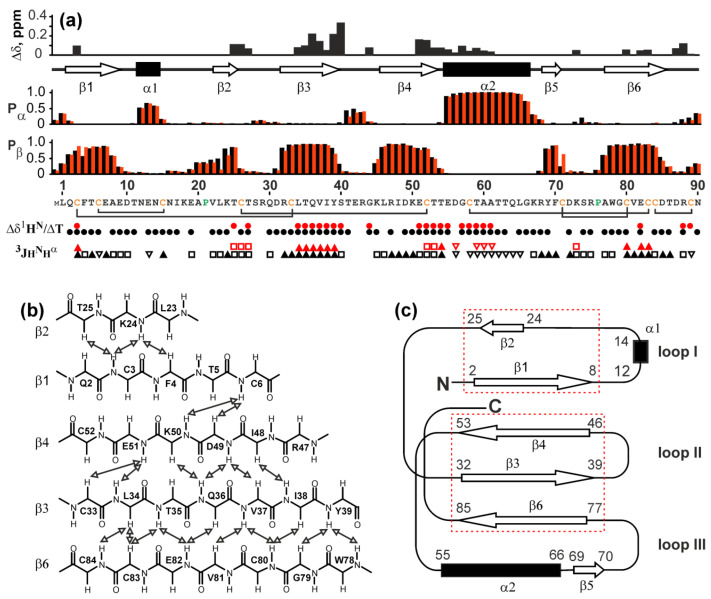
NMR data on secondary structure and conformational heterogeneity of Lystar5. Data for form I and form II are shown by black and red symbols, respectively. (**a**) **Δδ** – Difference in ^1^H^15^N chemical shifts of backbone amide groups between form I and form II (Δδ=(ΔδHN)2+(ΔδNH5)2); **Pα** and **Pβ**–Probabilities of α-helix and β-structure formation calculated from chemical shifts in the TALOS-N software; protein sequence is given in the bottom of the **Pβ** panel. Disulfide connectivities are shown by black brackets; the determined secondary structure is shown above the **Pα** panel; **Δδ^1^H^N^/ΔT**–temperature coefficients of amide protons. The filled circles denote amide protons with absolute values of temperature gradients less than 4.5 ppb/K; **^3^JH^N^H^α^**–coupling constants. The small (<5.5 Hz), large (>8.5 Hz), and medium (others) couplings are designated by open triangles, filled triangles and open squares, respectively; (**b**) Scheme of contacts between β-strands observed in the NOESY spectra. (**c**) Scheme of folding of secondary structure elements. β-Sheets formed by β1/ β2 and β3/β4/β6 strands are shown by red dotted rectangles.

Signal doubling was often observed in TFPs due to the *cis-trans* isomerization of the X-Pro peptide bonds [37]. However, in the case of Lystar5, the distribution of the difference in chemical shifts did not correlate with the location of two proline residues (Figure 6 and Figure S4a). This points to another source of conformational heterogeneity. However, without the experimentally determined spatial structure of Lystar5, it is impossible to establish the true cause of the conformational exchange.

The determined chemical shift values were used to calculate the secondary structure using the TALOS-N program [43]. The obtained data were also supported by the values of H^N^H^α^ scalar couplings as well as temperature gradients of amide protons (Figure 6a). Six β-strands were identified: Gln2-Ala8 (β1), Lys24-Thr25 (β2), Arg32-Tyr39 (β3), Leu46-Thr53 (β4), Tyr69-Phe70 (β5), and Ala77-Asp85 (β6). In addition, two helical segments were observed: short turn Asn12-Asn14 (α1) and distinct long α-helix Glu55-Gly66 (α2). The relative positions of the β-strands were determined from the contacts in the 3D ^15^N-NOESY-HSQC spectrum (mix 0.2s) and from the structural homology with other TFPs (Figure 6b). It was shown that the structure contains two antiparallel β-sheets: the first one contains the β1 and β2 strands, the second one includes β3, β4, and β6 strands. The strands β1 and β2 form the loop I, which also contains a short helical turn α1. The strands β3, β4, and β6 form the second β-sheet, which stabilizes the loops II and III. The loop III contains the α2 helix and additional short segment β5 (Figure 6c). Notably, the Gln2-Ala8 (β1) and Lys24-Thr25 (β2) segments that form the loop I have significantly different lengths, although demonstrate a high stability of the β-structure (Figure 6a). This can be explained by stabilization of the β1 segment not only by the contacts with the β2 segment, but also with the β4 strand from the adjacent β-sheet. This β1-β4 contact was confirmed by the cross-peaks in the NOESY spectrum (Figure 6b). Similarly, the short β5-segment (Tyr69-Phe70) in the loop III most likely interacts with the “outer” side of the second β-sheet in the region of the β6-strand. 

**Figure 7:**
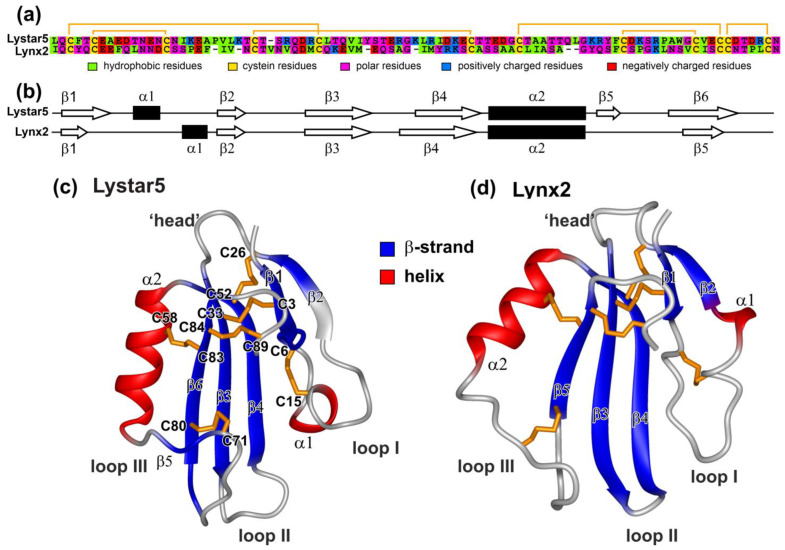
Comparison of structural features of Lystar5 and Lynx2. (**a**) Alignment of primary structures of Lystar5 and Lynx2. Disulfide bonds are shown by orange brackets. (**b**) Comparison of secondary structures of Lystar5 and Lynx2. (**c**) Ribbon representation of the Lystar5 spatial structure predicted by AlphaFold2 [45]. (**d**) Ribbon representation of the Lynx2 spatial structure obtained by NMR (PDB Id 6ZSS). Experimentally defined elements of the secondary structure are denoted by color: β-strands in blue and helical elements in red. Disulfide bonds are in gold.

In our work, we were not able to confirm the disulfide bond connection pattern in Lystar5 directly due to low intensity and overlapping of the NMR signals, which did not allow to observe H^β^-H^β^ NOE contacts between the cysteine residues. However, our previous results suggest that the three-finger fold could be formed only upon the proper disulfide connection [44]. Moreover, the formation of the three-finger fold suggests a single way to close the disulfide bonds. During refolding, different fractions of Lystar5 were formed and separated by HPLC (Figure S3), which apparently corresponded to differently closed disulfides. Since the fraction containing the correctly folded structure was selected using ^1^H NMR, our data on the three-finger structure of Lystar5 (Figure 6c) serve as indirect evidence that the disulfide bond pattern in the recombinant starfish protein corresponds to that observed in other TFPs (Figure 7). In total, the data obtained are consistent with the “three-finger” architecture of Lystar5 and prove the structural homology with the Ly6/uPAR proteins, and in particular, with the human brain neuromodulator Lynx2, also known as LYPD1 [37] (Figure 7). 

### 2.6. Study of Lystar5 Pharmacology at nAChRs

**Figure 8:**
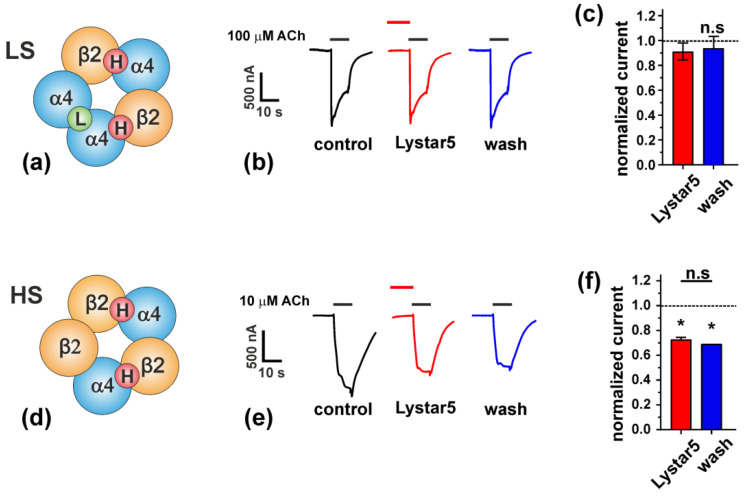
Lystar5 interaction with α4β2-nAChRs. (**a,d**) Composition of high selective (HS) and low selective (LS) subtypes of α4β2-nAChR. (**b,e**) Representative current traces through LS (**b**) and HS (**e**) α4β2-nAChRs expressed in *X. laevis* oocytes, evoked by 10 µM (HS receptors) or 100 µM (LS receptors) acetylcholine (ACh). The bars above the traces designate the application of specific compounds, the Lystar5 bar is off-scale along time axis (50 µM Lystar5 preincubation time 15s). (**c,f**) Average current amplitudes normalized to the control currents (before Lystar5 application) at LS (**c**) and HS (**f**) receptors, mean ± SEM, *n* = 4. * (*p* < 0.05) indicates significant differences between the group and control value (1.0) by one-sample two-sided *t*-test, n.s.—non-significant difference.

*A. rubens* protein Lystar5 has high degree of homology with the amino acid sequence (Figure 2) and 3D structure (Figure 7) of the mammalian neuromodulator Lynx2. The main known target of Lynx2 are the heteromeric α4β2-nAChRs [9]. It was shown previously that Lynx2 inhibits activation of this receptor [38]. Here, we studied the Lystar5 activity on the human receptor of this type expressed in *X. laevis* oocytes. Notably, due to heteromeric composition, α4β2-nAChR can be composed of three α4 and two β2 subunits (so called low selective channel, LS, Figure 8a) or from two α4 and three β2 subunits (so called high selective channel, HS, Figure 8d). We tested the Lystar5 activity on both forms of α4β2-nAChR (LS and HS). Previously described values of IC_50_ for human TFPs at different nAChRs expressed in oocytes lay in micromolar ranges. For example: IC_50_ of Lypd6 at α3β4-nAChR is ~40 µM [15], IC_50_ of Lynx1 at α7-nAChR is ~50 µM [46], and IC_50_ of SLURP-1 at α7-nAChR is ~10 µM [47]. Thus, we used here the single concentration of Lystar5 (50 µM).

Activation of α4β2-nAChRs by 10 or 100 µM acetylcholine (ACh) evoked currents through the oocyte’s membrane in the both cases (LS and HS receptor forms). At the same time, 15 s preincubation with 50 µM Lystar5 affected only the HS form of the receptor (Figure 8b,c,e,f). Lystar5 negatively modulated the HS α4β2-nAChRs with inhibition of current amplitude by ~30% and irreversible binding (Figure 8e,f). No effect of Lystar5 alone (in the absence of ACh) on the receptor activity was found. We tested the Lystar5 activity also on α3β2-nAChR and found no effect (data not shown). Thus, the starfish protein Lystar5 shares not only the primary and spatial structure with the human neuromodulator Lynx2, but also its pharmacology. 

### 2.7. Lystar5 Down-Regulates Expression of nAChRs and Acetylcholine Esterase

**Figure 9:**
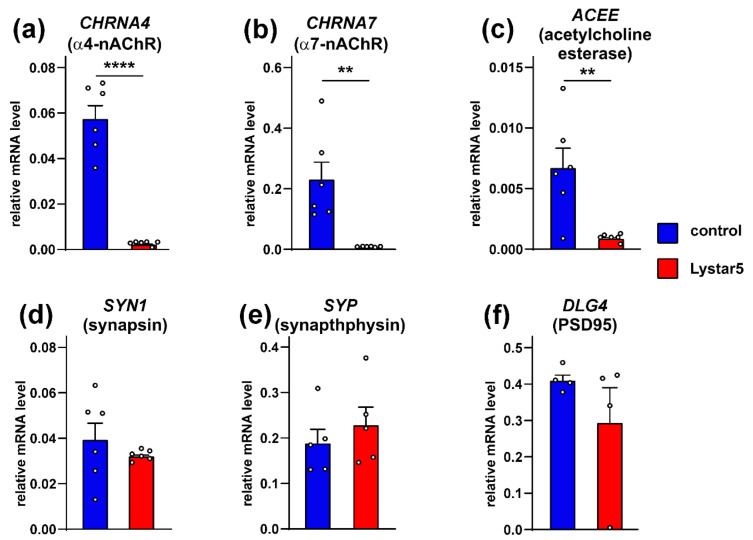
Analysis of the Lystar5 influence on expression of α4 (**a**), α7 (**b**) nAChR subunits, acetylcholine esterase (**c**), synapsin (**d**), synaptophysin (**e**), and PSD95 (**f**) in rat hippocampal neurons upon 7-day incubation. The mRNA level was normalized to the β-actin level and presented as a relative level ± SEM (*n* = 4–6). ** (*p* < 0.01) and **** (*p* < 0.0001) indicate significant difference between the data groups according to two-sided *t*-test.

Some mammalian TFPs play regulatory roles affecting not only the function of their target receptors, but also expression of the receptors and other proteins [48]. To establish whether Lystar5 can regulate the cholinergic system, we used hippocampal neurons as model cells and studied the expression of the α4 and α7 nAChR subunits, acetylcholine esterase, and factors important for synaptic plasticity (synapsin, synathophysin, and PSD95) by real-time PCR. Incubation of the neurons with Lystar5 dramatically reduced the expression of the nAChR subunits and acetylcholine esterase genes, while no influence on gene expression of the synaptic factors was found (Figure 9). 

## 3. Discussion

Last years, genome [31], transcriptomes of the nervous system [33], limbs (rays) [31], and gonads [32] and proteomes of the mucous secretion [31], nervous system [49,50], coelomocytes [51], and coelomic fluid [30] of the starfish *A. rubens* have been reported. In addition, genome, exoproteome, and transcriptomes of the eyes, nervous system, and reproductive system [34,35,36] of the starfish *A. planci* were published. Four TFPs (Lystar1–4) were identified in *A. rubens* [30], but their function was not studied yet. Here we analyzed available genomic data and found the set of putative TFPs in the both starfishes (Figure 1). Expression of one of them (Lystar5) in *A. rubens* coelomocytes was confirmed, and the three-finger structure of Lystar5 was proven by NMR (Figure 6 and Figure 7). 

The majority of found *A. rubens* proteins have orthologs in *A. planci* (Figure 4). This indicates that these proteins play an important regulatory role in the physiology of both starfish. Moreover, homologous TFPs of *A. rubens* and *A. planci* demonstrate significant sequence similarity with the LYPD6, LYPD6B, and Lynx2 (LYPD1) proteins from different species (Figure 2 and Figure 3). LYPD6 was found in many organisms from fish to human [11,13,22]; it is expressed in lung, kidneys, heart, liver, prostate, and brain [13,52], and is involved in the regulation of the cholinergic and Wnt signaling [11,15,22]. The function of LYPD6B is not well-studied, although it has high degree of the sequence similarity with LYPD6, so we can propose a similar function. In contrast, little is known about the biological role and function of Lynx2. At present, Lynx2 was reported only in mammals, and no expression patterns were determined, except for the brain regions [9]. It was also reported that Lynx2 increases anxiety-related behaviors and targets α4β2-nAChRs [38]. Therefore, it is surprising to find the Lynx2 homolog, - Lystar5-, in such “primitive” animal as starfish. This indicates the extreme importance of LYPD6 and Lynx2 orthologs and their involvement in vital processes that were retained in the course of evolution. It will be very interesting to follow the Lynx2 expression patterns and function in organisms from different taxonomic groups.

It is known that the molecular targets of LYPD6 and Lynx2 include different subtypes of nAChR [8,9,15]. Consistent with this, the starfish homologue of Lynx2 - Lystar5 - targets high selective (HS) α4β2-nAChRs (Figure 8). α4β2-nAChRs differ by distribution of ligand-binding sites located at the interfaces of adjacent subunits. HS channels contain two high affinity sites located at the interfaces of α4/β2 subunits (Figure 8d, [53]). At the same time, low selective (LS) channels contain three ligand-binding sites: one low affinity site at the α4/α4 interface and two high affinity α4/β2 sites (Figure 8a). Lystar5 most likely recognizes α4/β2 sites of the HS receptors, but the reason why it does not interact with the α4/β2 sites at the LS receptors remains unclear. Evidently, additional experiments are required to study the effect of Lystar5 on the α4β2 and other nAChR subtypes in details. α4β2-nAChR is one of the most abundant nicotinic receptor in the mammalian brain [54]. The targeting of this receptor possibly points to the involvement of Lystar5 in the neuronal regulation in starfish. Indeed, echinoderms possess cholinergic neuromuscular transmission, with excitatory nAChRs identified pharmacologically [55]. 

nAChRs are expressed not only in the nervous system, but also in non-neuronal tissues such as the epithelium [56] and immune system [57,58]. In higher animals the epithelial cholinergic system control cellular homeostasis. In mammals, α7- and α4β2-nAChRs are involved in the control of numerous processes including inflammation, gene transcription, proliferation, migration, etc. [57,59]. In addition, the acetylcholine inactivation by acetylcholine esterase may also enhance inflammatory processes [60]. The observed influence of Lystar5 on expression of the α7 and α4 nAChRs subunits and acetylcholine esterase (Figure 9) points to the possible Lystar5 participation in the similar processes in the starfish. Thus, another possible function of Lystar5 and other starfish proteins found here is the regulation of the cholinergic system in non-neuronal organs and control of tissue homeostasis and inflammation. In accordance with this assumption, Lystar5 expression was found in coelomocytes (Figure S1). Coelomic cells are implicated in the immune response, nutrient transport, pathogen elimination, phagocytosis, and formation of cellular clots to avoid the coelomic fluid loss [61,62,63]. The Lystar5 function in these cells may be connected with the clearance of durable foreign bodies, immune response, or the regeneration of coelomic epithelium, which is the possible source of coelomocytes [62]. Coelomocytes are able to proliferate and migrate, and may be considered “stem”-like cells in *A. rubens* [64,65]. Thus, Lystar5 from the coelomic cells may also be implicated in the starfish regeneration processes and coelomocytes replenishment.

The present search of TFPs in the starfish genomes using the known LU-domain sequences as a bait revealed five and six proteins in *A. rubens* and *A. planci*, respectively. These putative TFPs demonstrate moderate homology to the *A. rubens* TFPs identified previously [30] (pairwise similarity 37–59%, Figure S5, Tables S7 and S8). The employed search procedure obviously has limitations; therefore, it cannot fish out all TFP sequences from the genomes. There are most likely many more unidentified TFPs in the starfish. 

## 4. Materials and Methods

### 4.1. Prediction of TFPs from Starfishes

To find possible TFPs among the starfish genomes, we initially built a reference list of the known and characterized TFPs. To do this, sequences of proteins containing the LU-domain were extracted from UniProt database (EMBL-EBI, Hinxton, UK) [66], from which sequences no longer than 190 residues were selected to exclude proteins with more than one LU-domain. From the resulting list (153 proteins, Table S1), the sequences of LU-domains were isolated and were used for the search. The search for TFPs was carried out in a database of starfish proteins, which were built from available genome assembly (Accession codes: GCA_902459465.3 for *A.rubens* and GCA_001949145.1 for *A. planci*). In total, the search was carried out among 24,050 sequences for *A. rubens* and 32,215 sequences for *A. planci*. For TFPs search, BLASTP ver. 2.13.0 (NCBI, Bethesda, MD, USA) was used [67], taking e-value < 10^−5^ as the threshold.

From the found proteins, LU domains were isolated and combined with a list of reference LU-domains. The resulting sequences were subjected to multiple sequence alignment (MSA) using ClustalW ver. 2.1 (EMBL-EBI, Hinxton, UK) [68]. In this case, a modified substitution matrix was used: similar to BLOSUM62, in which a high (99) and a low (−4) coefficients were assigned for cysteine-cysteine substitutions and for cysteine substitutions for other amino acid residues, respectively. The guide tree obtained from MSA was used to visualize the homology between the sequences. The iTOL ver 6.0 service (Biobyte Solutions, Heidelberg, Germany) [69] was used to build a tree. To calculate pairwise similarity between sequences, the residues were divided into groups: hydrophobic (A,F,H,I,L,M,P,V,W), cysteines (C), polar (G,N,Q,S,T,Y), positively (K,R) and negatively (D,E) charged. Similarity was calculated as the percentage of coincidences of residues belonging to the same group in each position when comparing aligned sequences.

For prediction of the signal peptide and glycosylphosphatidylinositol (GPI) anchoring site in the Lystar5 sequence, SignalP ver. 6.0 (Department of Health Technology, Technical University of Denmark, Kongens Lyngby, Denmark) [70] and PredGPI ver. 1.0 (Biocomputing Group, Department of Biology, University of Bologna, Bologna, Italy) [71] web-services were used.

### 4.2. Animals and Tissue Isolation

Adult individuals of the starfish *A. rubens Linnaeus* 1758 were collected at the Biological Station of the Zoological Institute, Russian Academy of Sciences, on Cape Kartesh (Kandalaksha Bay, White Sea) in September 2018. Circulatory coelomocytes were collected by cutting off of an arm tip and draining the CF into a 50 mL tube containing 5 mL of Ca^2+^- and Mg^2+^-free saline solution [72] supplemented with 150 mM EDTA. The cells were pelleted by centrifugation at 120 g for 10 min in a bucket rotor, and snap-frozen in liquid nitrogen.

### 4.3. Confirmation of Lystar5 Expression in A. rubens

To confirm the expression of Lystar5 mRNA in *A. rubens*, we performed real-time PCR with two types of primers for *A. rubens* gene XM_033772626.1 (see Figure S1 for details). To perform the PCR experiments, the total RNA was isolated using the Bio-Rad Aurum RNA isolation kit (Bio-Rad, Hercules, CA, USA) according to the manufacturer instructions. cDNA was synthesized by the Mint reverse transcriptase kit (Evrogen, Moscow, Russia). After that, qPCR was performed with the ready to use SYBR Green HS mix (Evrogen, Moscow, Russia) and primers specific to the XM_033772626.1 (first pair: forward: CGGTGGGGAAAACAAACTGAC, reverse: ACGCTGTTCGAAAGCAGACT, amplicon length 184 b.p.; second pair: forward: GCTCCGGTTCTCAAGACGTG, reverse: GTATCGCAGCACTCGACACA, amplicon length 200 b.p.) using the Roche LightCycler 96 amplifier (Roche, Basel, Switzerland). Data were analyzed qualitatively using the LightCycler SW software (Roche, Basel, Switzerland).

### 4.4. Design of the Lystar5 Gene for the Recombinant Production

The gene for the recombinant production of Lystar5 was created based on the amino acid sequence XP_033628517.1 from the NCBI database (residues 38–137) and optimized for the codon usage frequency in *E. coli*. The gene was cloned into *pET-22b(+)* vector. A start codon atg, encoding a methionine residue, was added to the 5’ end of the gene. 

### 4.5. Bacterial Production of Lystar5 

For the production of Lystar5, *E. coli* expression strain BL21(DE3) was used. Transformed cells were grown at 37 °C on TB bacterial growth media. Expression of Lystar5 was induced by addition of Isopropyl β-d-1-thiogalactopyranoside to a final concentration of 0.05 mM at A_600_ of 0.6. Extraction of the target protein from the inclusion bodies was performed under denaturing conditions (8 M urea). In order to increase the efficiency of ion exchange chromatography purification, Cys residues in the Lystar5 molecule were chemically modified to S-sulfites (Cys-S-SO_3_) using sodium tetrathionate and sodium sulfite. After chromatography, sulfo-groups were cleaved by an excess of dithiothreitol (DTT). For purification of reduced Lystar5, reverse-phase HPLC was performed (10 × 250 mm, A300, Jupiter, Phenomenex, Torrance, CA, USA), then the fractions containing Lystar5 were lyophilized. Refolding was performed by dissolving reduced Lystar5 to a final concentration of 0.01 mg/mL in a renaturation buffer (50 mM Tris/HCl, 1.5 M urea, 0.5 M l-arginine, 0.1 M NaCl, 4 mM GSH, 1 mM GSSG, pH 9.0) and incubation during 3 days at 4 °C. After renaturation, the protein was purified on reverse-phase C4 HPLC column (4.6 × 250 mm, A300, Jupiter, Phenomenex, Torrance, CA, USA). 

For production of 13C, 15N-labled Lystar5, transformed cells were grown on LB bacterial growth media. When the culture reached A_600_ of 0.6, the cells were harvested (2000 g for 20 min) and transferred into bioreactor (Sartorious, Goettingen, Germany) containing M9 minimal medium with 10% of thiamine chloride and ^15^NH_4_Cl and ^13^C-glucose as sources of nitrogen and carbon. Afterward, gene expression was induced.

### 4.6. Assignment of ^13^C-^15^N-NMR Spectra of Lystar5 and Secondary Structure Determination

NMR experiments were done using 0.04 mM sample of ^13^C,^15^N-labled Lystar5 in H_2_O solution with 5% D_2_O at pH 7.0 and 37 °C. NMR spectra were acquired on a Bruker Avance 800 spectrometer (Billerica, MA, USA) equipped with cryoprobe. ^1^H, ^13^C, and ^15^N resonance assignment was obtained by a standard procedure using combination of 2D and 3D spectra [73]. The ^3^J_HNHα_ coupling constants were determined using the 3D HNHA experiment [74]. Prediction of the secondary structure was performed using the TALOS-N ver. 4.12 software (Ad Bax group, NIDDK, NIH, Bethesda, MD, USA) [43]. Temperature gradients of amide protons were measured in the 2D ^15^N-HSQC spectra obtained in the temperature range 15–45 °C with 5 °C step. 

For visualization, a model of spatial structure of Lystar5 LU-domain was built using AlphaFold ver. 2.1.0 Collab notebook (EMBL-EBI, Hinxton, UK) [45]. 

### 4.7. Electrophysiological Recordings in X. laevis Oocytes

Human nicotinic acetylcholine receptors (α4)_3_(β2)_2_ (low sensitive to nicotine, α4β2 LS), (α4)_2_(β2)_3_ (high sensitive to nicotine, α4β2 HS) were expressed in *X. laevis* oocytes, which were prepared and injected as described previously [46]. For α4β2 LS and α4β2 HS mRNA of individual subunits were injected at 10:1 and 1:10 α4:β2 molar ratio, respectively. Plasmids encoding α4 and β2 nAChR subunits were kindly provided by Prof. P.-J. Corringer.

Two-electrode voltage clamp recordings were done as described previously [46]. The oocytes were continuously perfused with ND-96 solution (96 mM NaCl, 2 mM KCl, 1.8 mM CaCl_2_, 1 mM MgCl_2_, 10 mM HEPES, pH 7.4) at 2.5 mL/min flow rate. Currents were elicited by application of acetylcholine solution in ND-96 (100 uM for α4β2 LS and 10 uM for α4β2 HS) for 10 s with 5 min intervals between applications. If needed, 50 µM Lystar5 was applied by pre-incubation with ND-96 solution containing the protein for 15 s before acetylcholine application. The 10 mM stock of Lystar5 in 100% DMSO was diluted to target concentrations with ND-96 right before experiment. Current responses under Lystar5 treatment were normalized to control responses to acetylcholine with pre-incubation in ND-96 solution without the protein for the same oocyte. 

### 4.8. Primary Neuron Culture 

All animal care and experimental procedures were performed in accordance with the guidelines set forth by the European Communities Council Directive of November 24, 1986 (86/609/EEC) and were approved by the Ethical Committee of the Shemyakin-Ovchinnikov Institute of Bioorganic Chemistry RAS for the control of the maintenance and use of animals (protocol #312 from 18 December 2022).

The primary cultures of neurons from the hippocampus were obtained as previously described [75]. Briefly, new-born rat pups were anesthetized, decapitated, and the hippocampus was isolated, homogenized by scalpel and incubated 15 min in 0.8% trypsin solution in the DME medium. After that, the homogenate was centrifuged at 500 g for 2 min. The sediment was suspended in the Neurobasal-A medium (Gibco, Whaltham, MA, USA) and dissociated by aspiration through a flame-polished 1 ml pipette repeated five times. Then, neurons were seeded on poly(l)-Lysine-coated glasses in 24-well plates, and the medium was changed after 1-h incubation in a humidified atmosphere. To inhibit a growth of glial cells, 20 μM Cytarabine (Sigma-Aldrich, St. Louis, MO, USA) was added on the third day of cultivation. Neurons were cultivated for 12 days with a medium change every 4 days. At day 21, the neurons were incubated with 10 µM of Lystar5 from 10 mM DMSO stock or equal DMSO amount for 7 days. Thereafter the real-time PCR experiments were carried out.

### 4.9. Real-Time PCR

Total RNA was isolated using the Bio-Rad Aurum RNA isolation kit (Bio-Rad, Hercules, CA, USA) according to the manufacturer instructions. cDNA was synthesized by the Mint reverse transcriptase kit (Evrogen, Moscow, Russia). After that, qPCR was performed with the ready to use SYBR Green HS mix (Evrogen, Moscow, Russia) and primers specific to the *CHRNA4*, *CHRNA7*, *ACEE*, *SYN1*, *SYP*, and *DLG4* (Table S9) using the Roche LightCycler 96 amplifier (Roche, Basel, Switzerland). Data were analyzed by the ∆∆Ct method and LightCycler SW software (Roche, Basel, Switzerland), and the gene expression was normalized to the expression of *β-ACTIN*, housekeeping gene.

## 5. Conclusions

Here, for the first time, we reported the starfish three-finger protein Lystar5 sharing the structure and pharmacology with the mammalian neuromodulator Lynx2. The set of other starfish TFPs homologous to the Lynx2, LYPD6, and LYPD6B proteins were predicted. We propose that Lystar5 is the modulator of the cholinergic system in starfish and may serve for regulation of neuromuscular transmission or coelomocytes homeostasis. 

## Data Availability

Data generated within experiments is available on request.

## Supplementary Materials

The following supporting information can be downloaded at: https://www.mdpi.com/article/10.3390/md20080503/s1, Figure S1: The primer design and analysis for investigation of Lystar5 expression in *A.rubens*; Figure S2: Full sequence of Lystar5 from *A. rubens* (XP_033628517.1/LyAr1) [70,71]; Figure S3: Characterization of the recombinant Lystar5 protein; Figure S4: Data on two structural forms of Lystar5; Figure S5: Multiple sequence alignment of TFPs from *Asterias rubens* found by BLAST search and identified early by proteomics [30]; Table S1: List of known Ly6/uPAR proteins which were used for BLAST search of TFP in the starfishes; Table S2: List of putative LU-domain containing proteins which were identified in the genomes of starfishes; Table S3–S5: Pairwise similarity between some TFPs from starfishes and previously known TFPs; Table S6: Chemical shifts of the signals of backbone atoms Lystar5 in H_2_O solution; Table S7: Pairwise similarity calculated for TFPs from *A. planci*, identified by BLAST search, and previously identified TFPs from *A. rubens* (Lystar1-4) [30]; Table S8: Pairwise similarity calculated for TFPs from *A. rubens*, identified by BLAST search and previously identified TFPs from *A. rubens* (Lystar1-4); Table S9: Primers used for qPCR experiments.
