# Supplementary material for: New Three-Finger Protein from Starfish Asteria rubens Shares Structure and Pharmacology with Human Brain Neuromodulator Lynx2
Source: Mar Drugs. 2022 Aug 3;20(8):503. doi: 10.3390/md20080503 (PMC9410279; doi:10.3390/md20080503)
Supplement: Supplementary file 1 [file marinedrugs-20-00503-s001.zip › marinedrugs-1832311-supplementary.pdf]

marine drugs

MDPI

Supplementary material

Article

New Three-Finger Protein from Starfish *Asteria rubens* Shares Structure and Pharmacology of Human Brain Neuromodulator Lynx2

Alexander S. Paramonov[1], Mikhail A. Shulepko[1], Alexey M. Makhonin[1,2], Maxim L. Bychkov[1,] Dmitrii S. Kulbatskii[1], Andrey M. Chernikov[1,3], Mikhail Yu. Myshkin[1], Sergey V. Shabelnikov[4], Zakhar O. Shenkarev[1,5], Mikhail P. Kirpichnikov[1,3] and Ekaterina N. Lyukmanova[1,3,5,*]

[1] Shemyakin-Ovchinnikov Institute of Bioorganic Chemistry, Russian Academy of Sciences, Miklukho-Maklaya str. 16/10, 119997, Moscow, Russia; apar@nmr.ru (A.S.P.); mikhailshulepko@gmail.com (M.A.S.); ammakhonin@edu.hse.ru (A.M.M.); maksim.bychkov@gmail.com (M.L.B.); d.kulbatskiy@gmail.com (D.S.K.); chernikov.andrei.m@gmail.com (A.M.C.); mikhail.myshkin@phystech.edu (M.Y.M); zakhar-shenkarev@yandex.ru (Z.O.S.); kirpichnikov@inbox.ru (M.P.K.); ekaterina-lyukmanova@yandex.ru (E.N.L.)

[2] AI centre, National Research University Higher School of Economics, Myasnitskaya str. 20, 101000, Moscow, Russia

[3] Interdisciplinary Scientific and Educational School of Moscow University «Molecular Technologies of the Living Systems and Synthetic Biology», Faculty of Biology, Lomonosov Moscow State University, Leninskie Gory, 119234, Moscow, Russia.

[4] Institute of Cytology of the Russian Academy of Sciences, Tikhoretsky prospect 4, 194064, St. Petersburg, Russia; buddasvami@gmail.com (S.V.S.)

[5] Moscow Institute of Physics and Technology (State University), Institutskiy per. 9,141701, Dolgoprudny, Moscow Region, Russia.

* Correspondence: ekaterina-lyukmanova@yandex.ru

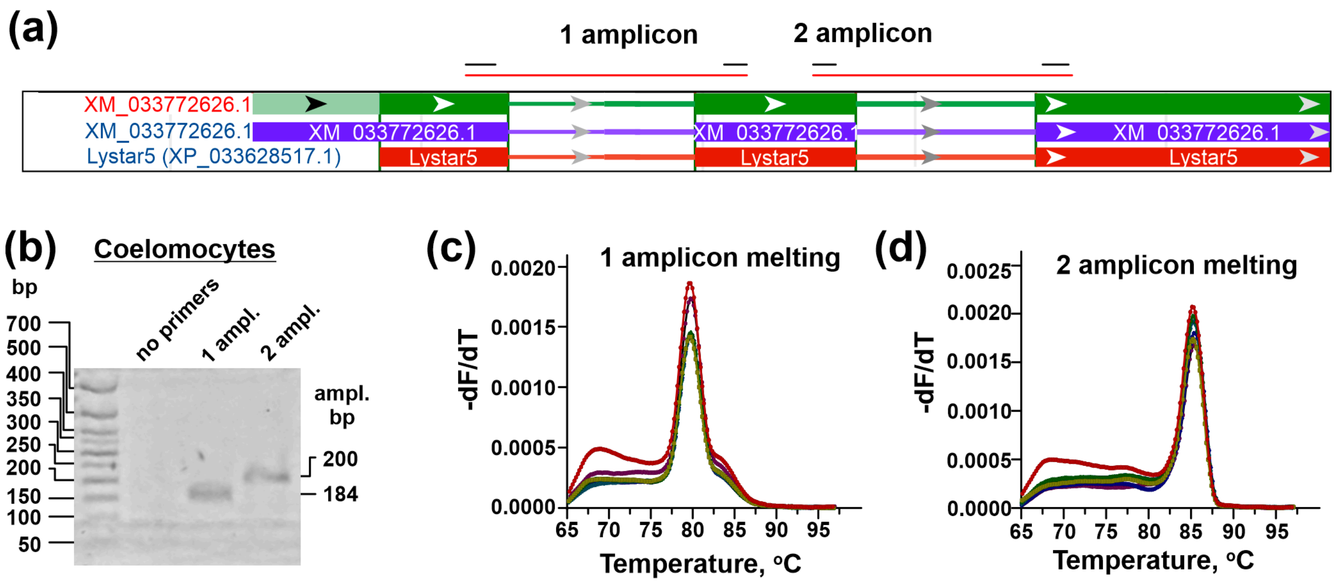

Figure S1. The primer design and analysis of Lystar5 expression in *A. rubens*. (a) The primer design scheme, the amplicons 1 and 2 are shown by red lines. **(b)** Representative electrophoretic analysis of the product obtained after the PCR reaction with negative control (no primers in the reaction) and in the presence of the primer pair 1 and 2 with cDNA etracted from coelomocytes. The product length is shown on the right. **(c,d)** the melting curves of amplicons 1 **(c)** and 2 **(d)** obtained during real-time PCR.

```
              signal peptide                         LU-domain
        10         20         30         40         50         60
MRIKMVFKFR RNSEGIFCIL MTLMIILMAV CFRTASSLQC FTCEAEDTNE NCNIKEAPVL
        70         80         90        100        110        120
KTCTSRQDRC LTQVIYSTER GKLRIDKECT TEDGCTAATT QLGKRYFCDK SRPAWGCVEC
       130        140        150
CDTDRCNENG VAVTRASAAV VLGALSSVVV ALHVL
     GPI site                removed in mature form
```

Figure S2. Full sequence of Lystar5 from *A. rubens* (XP_033628517.1/LyAr1). Signal peptide predicted by SignalP [1] and the glycosylphosphatidylinositol (GPI) anchoring site predicted by PredGPI [2] are shown by red and blue, respectively.

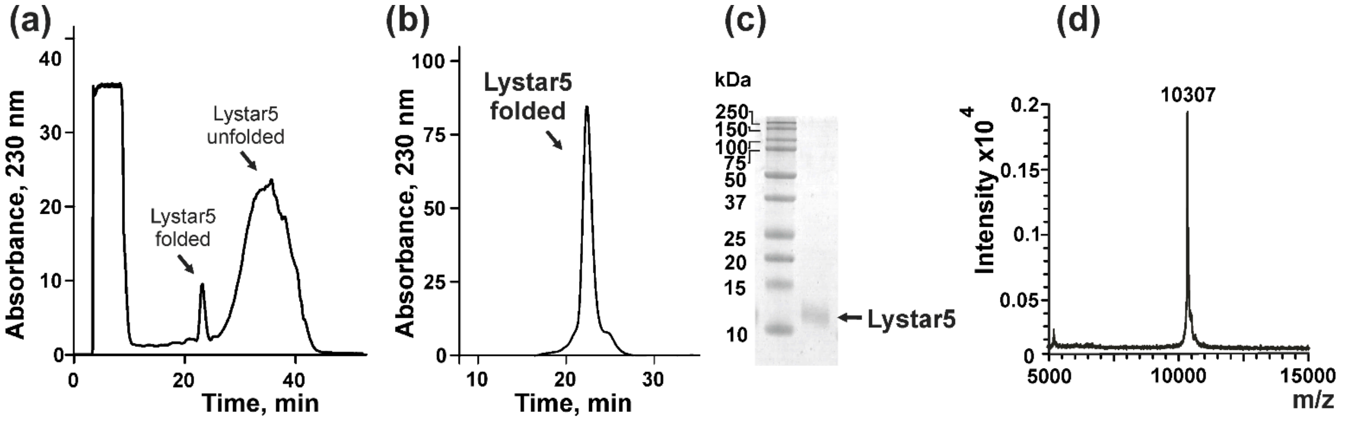

Figure S3. Characterization of the recombinant Lystar5 protein. (a) Representative chromatogram of Lystar5 HPLC purification. Fractions corresponding to the folded and unfolded Lystar5 are shown. **(b)** Analytical HPLC of folded Lystar5. **(c)** SDS-PAGE analysis of folded Lystar5. **(d)** Mass-spectrometry analysis of folded Lystar5.

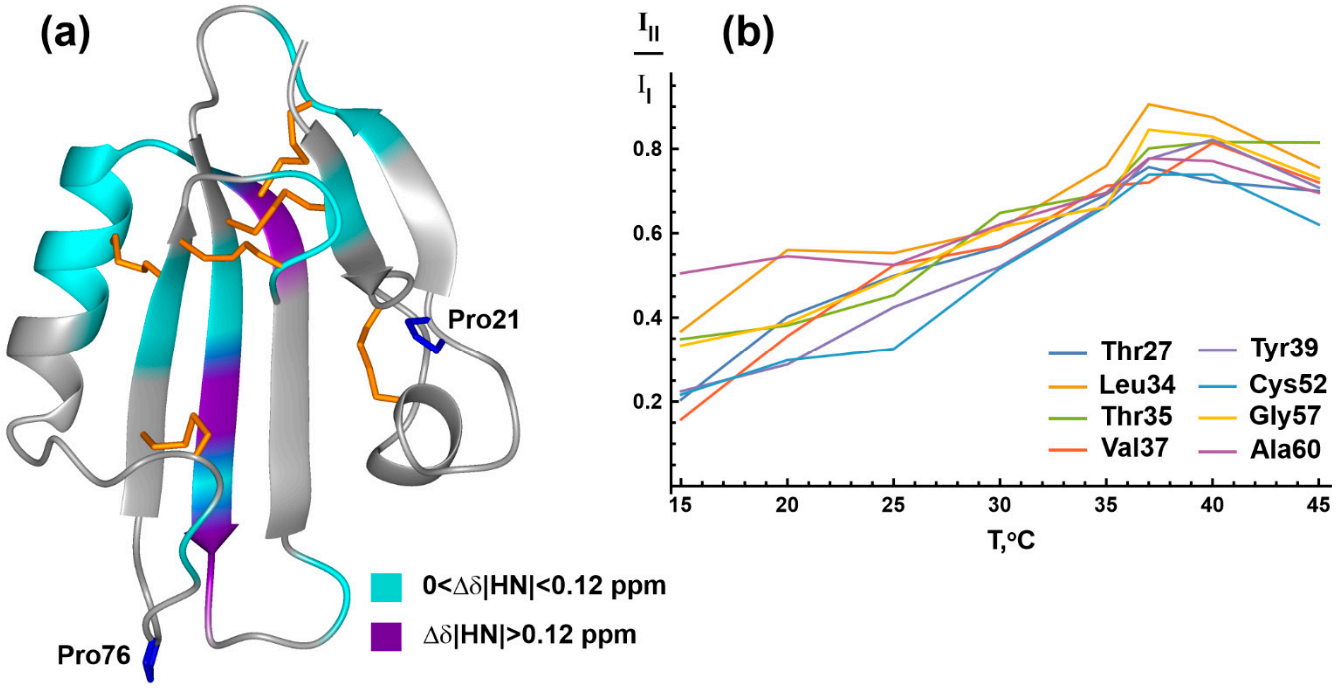

Figure S4. Data on two structural forms of Lystar5. **(a)** Ribbon representation of 3D structure of Lystar5 obtained using AlphaFold2. Colors show residues for which there was a difference in the chemical shifts of amide groups between the two forms ($\Delta\delta|HN| = \sqrt{(\Delta\delta_{HN})^2 + (^{\Delta\delta_{NH}}/_{5})^2}$). Residues with the greatest difference (Δδ|HN|>0.12ppm) are marked in purple. Sidechains of prolines are shown in blue. Disulfides are shown in gold. **(b)** Temperature dependence of ratio of signal intensities in the ^{15}N-HSQC spectra for the form II and form I for some residues.

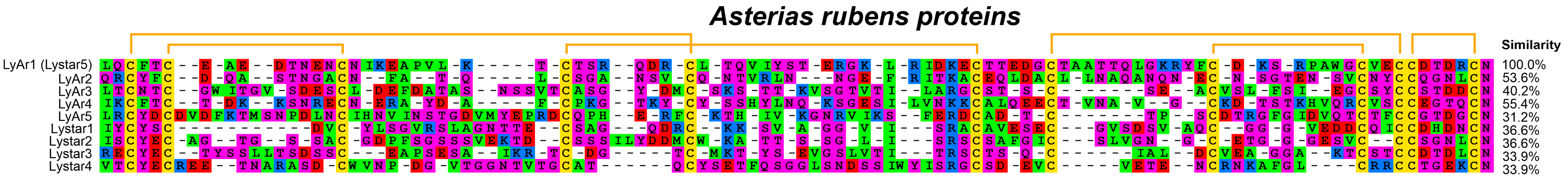

Figure S5. Multiple sequence alignment of TFPs from *Asterias rubens* found by BLAST search (LyAr1-5) and identified early by proteomics (Lystar1-4) [3].

Table S1. List of known Ly6/uPAR proteins which were used for BLAST search of TFP in starfishes.

No.	Name	Description	Organism	Access code
**Snake toxins**				
1	aBTX	alpha-bungarotoxin	*Bungarus multicinctus*	P60615
2	alpha cobratoxin	alpha-cobratoxin	*Naja kaouthia*	P01391
3	LSTIII	alpha-elapitoxin-Ls2a	*Laticauda semifasciata*	P01379
4	long neurotoxin1	alpha-elapitoxin-Nno2a	*Naja oxiana*	P01382
5	bCTX	beta-cardiotoxin CTX27	*Ophiophagus hannah*	Q69CK0
6	bucandin	aucandin	*Bungarus candidus*	P81782
7	calciseptine	calciseptin	*Dendroaspis polylepis polylepis*	P22947
8	candoxin	candoxin	*Bungarus candidus*	P81783
9	cobrotoxin	cobrotoxin	*Naja atra*	P60770
10	cytotoxin1	cytotoxin 1	*Naja kaouthia*	P60305
11	cytotoxin2	cytotoxin 2	*Naja kaouthia*	P01445
12	cytotoxin3	cytotoxin 3	*Naja kaouthia*	P01446
13	dendroaspin	dendroaspin	*Dendroaspis jamesoni kaimosae*	P28375
14	denmotoxin	denmotoxin	*Boiga dendrophila*	Q06ZW0
15	erabutoxin a	erabutoxin a	*Laticauda semifasciata*	P60775
16	erabutoxin b	erabutoxin b	*Laticauda semifasciata*	Q90VW1
17	fasciculin-1	Fasciculin-1	*Dendroaspis angusticeps*	P0C1Y9
18	fasciculin-2	Fasciculin-2	*Dendroaspis angusticeps*	P0C1Z0
19	haditoxin	Haditoxin	*Ophiophagus hannah*	A8N286
20	irditoxin	irditoxin subunit A	*Boiga irregularis*	A0S864
21	kappaBTX	kappa-bungarotoxin	*Bungarus multicinctus*	P01398
22	mambalgin1	mambalgin-1	*Dendroaspis polylepis polylepis*	P0DKR6
23	mambalgin2	mambalgin-2	*Dendroaspis polylepis polylepis*	P0DKS3
24	mambalgin3	mambalgin-3	*Dendroaspis angusticeps*	C0HJB0
25	micrurotoxin-1	micrurotoxin 1	*Micrurus mipartitus*	C0HJR1
26	micrurotoxin2	micrurotoxin 2	*Micrurus mipartitus*	C0HJR2
27	MT1	Muscarinic toxin 1	*Dendroaspis angusticeps*	P81030
28	MT2	Muscarinic toxin 2	*Dendroaspis angusticeps*	P18328
29	MT3	Muscarinic toxin 3	*Dendroaspis angusticeps*	P81031
30	MT4	Muscarinic toxin 4	*Dendroaspis angusticeps*	Q9PSN1
31	MT7	Muscarinic toxin 7	*Dendroaspis angusticeps*	Q8QGR0
32	alpha short neurotoxin 1	Short neurotoxin 1	*Naja pallida*	P01426
33	short neurotoxin 1	Short neurotoxin 1	*Naja oxiana*	P01427
34	S2C4	Synergistic-type venom protein S2C4	*Dendroaspis jamesoni kaimosae*	P01407
35	3FTX	Three-finger toxin MALT0070C	*Micrurus altirostris*	F5CPE6
36	FS2	Toxin FS-2	*Dendroaspis polylepis polylepis*	P01414
37	LC	Toxin Lc b	*Laticauda colubrina*	P0C8R8
38	S4C8	Toxin S4C8	*Dendroaspis jamesoni kaimosae*	P25683
39	WTX	Tryptophan-containing weak neurotoxin	*Naja kaouthia*	P82935
**Mammalia**				

No.	Name	Description	Organism	Access code
40	CD59	CD59 glycoprotein	*Oryctolagus cuniculus*	O77541
41	CD59	CD59 glycoprotein	*Papio sp.*	Q28785
42	CD59	CD59 glycoprotein	*Callithrix sp.*	P46657
43	CD59	CD59 glycoprotein	*Rattus norvegicus*	P27274
44	CD59	CD59 glycoprotein	*Chlorocebus aethiops*	Q28216
45	CD59	CD59 glycoprotein	*Sus scrofa*	O62680
46	CD59	CD59 glycoprotein	*Macaca fascicularis*	Q8SQ46
47	CD59	CD59 glycoprotein	*Homo sapiens*	P13987
48	CD59	CD59 glycoprotein	*Saimiri sciureus*	P47777
49	CD59	CD59 glycoprotein	*Aotus trivirgatus*	P51447
50	CD59	CD59 glycoprotein	*Pongo abelii*	Q5R510
51	CD59	CD59A glycoprotein	*Mus musculus*	O55186
52	CD59B	CD59B glycoprotein	*Mus musculus*	P58019
53	GPIHBP1	Glycosylphosphatidylinositol-anchored high density lipoprotein-binding protein 1	*Homo sapiens*	Q8IV16
54	GML	Glycosyl-phosphatidylinositol-anchored molecule-like protein	*Homo sapiens*	Q99445
55	LYNX1	Ly-6/neurotoxin-like protein 1	*Macaca mulatta*	P0DP62
56	LYNX1	Ly-6/neurotoxin-like protein 1	*Bos taurus*	Q1RMQ4
57	LYNX1	Ly-6/neurotoxin-like protein 1	*Homo sapiens*	P0DP58
58	LYNX1	Ly-6/neurotoxin-like protein 1	*Pan troglodytes*	Q5IS42
59	LYNX1	Ly-6/neurotoxin-like protein 1	*Saimiri boliviensis boliviensis*	Q5IS87
60	LYNX1	Ly-6/neurotoxin-like protein 1	*Mus musculus*	P0DP60
61	LYPD1	Ly6/PLAUR domain-containing protein 1	*Rattus norvegicus*	Q66H42
62	LYPD1	Ly6/PLAUR domain-containing protein 1	*Homo sapiens*	Q8N2G4
63	LYPD1	Ly6/PLAUR domain-containing protein 1	*Mus musculus*	Q8BLC3
64	LYPD2	Ly6/PLAUR domain-containing protein 2	*Homo sapiens*	Q6UXB3
65	LYPD2	Ly6/PLAUR domain-containing protein 2	*Mus musculus*	Q9DD23
66	LYPD6	Ly6/PLAUR domain-containing protein 6	*Homo sapiens*	Q86Y78
67	LYPD6	Ly6/PLAUR domain-containing protein 6	*Rattus norvegicus*	D3ZTT2
68	LYPD6	Ly6/PLAUR domain-containing protein 6	*Mus musculus*	Q8BPP5
69	LYPD6B	Ly6/PLAUR domain-containing protein 6B	*Mus musculus*	Q9D7F2
70	LYPD6B	Ly6/PLAUR domain-containing protein 6B	*Homo sapiens*	Q8NI32
71	LY6G5C	Lymphocyte antigen 6 complex locus protein G5c	*Rattus norvegicus*	Q8CHN2
72	LY6G5C	Lymphocyte antigen 6 complex locus protein G5c	*Macaca mulatta*	Q863H0
73	LY6G5C	Lymphocyte antigen 6 complex locus protein G5c	*Mus musculus*	Q8K1T5

No.	Name	Description	Organism	Access code
74	LY6G5C	Lymphocyte antigen 6 complex locus protein G5c	*Canis lupus familiaris*	Q9XSV5
75	LY6G5C	Lymphocyte antigen 6 complex locus protein G5c	*Homo sapiens*	Q5SRR4
76	LY6G6C	Lymphocyte antigen 6 complex locus protein G6c	*Homo sapiens*	O95867
77	LY6G6C	Lymphocyte antigen 6 complex locus protein G6c	*Mus musculus*	Q9Z1Q4
78	LY6G6C	Lymphocyte antigen 6 complex locus protein G6c	*Bos taurus*	A0JNL5
79	LY6G6D	Lymphocyte antigen 6 complex locus protein G6d	*Homo sapiens*	O95868
80	LY6G6D	Lymphocyte antigen 6 complex locus protein G6d	*Rattus norvegicus*	Q6MG58
81	LY6G6D	Lymphocyte antigen 6 complex locus protein G6d	*Mus musculus*	Q9Z1Q3
82	LY6A	Lymphocyte antigen 6A-2/6E-1	*Mus musculus*	P05533
83	LY6B	Lymphocyte antigen 6B	*Rattus norvegicus*	Q63317
84	LY6C1	Lymphocyte antigen 6C1	*Mus musculus*	P0CW02
85	LY6C2	Lymphocyte antigen 6C2	*Mus musculus*	P0CW03
86	LY6D	Lymphocyte antigen 6D	*Homo sapiens*	Q14210
87	LY6D	Lymphocyte antigen 6D	*Mus musculus*	P35459
88	LY6D	Lymphocyte antigen 6D	*Bos taurus*	Q148C3
89	LY6E	Lymphocyte antigen 6E	*Homo sapiens*	Q16553
90	LY6E	Lymphocyte antigen 6E	*Mus musculus*	Q64253
91	LY6F	Lymphocyte antigen 6F	*Mus musculus*	P35460
92	LY6G	Lymphocyte antigen 6G	*Mus musculus*	P35461
93	LY6G6E	Lymphocyte antigen 6G6e	*Mus musculus*	Q8K1T6
94	LY6H	Lymphocyte antigen 6H	*Macaca fascicularis*	Q4R5M8
95	LY6H	Lymphocyte antigen 6H	*Bos taurus*	A0JNB3
96	LY6H	Lymphocyte antigen 6H	*Homo sapiens*	O94772
97	LY6H	Lymphocyte antigen 6H	*Mus musculus*	Q9WUC3
98	LY6I	Lymphocyte antigen 6I	*Mus musculus*	Q9WU67
99	LY6K	Lymphocyte antigen 6K	*Homo sapiens*	Q17RY6
100	LY6K	Lymphocyte antigen 6K	*Mus musculus*	Q9CWP4
101	LY6L	Lymphocyte antigen 6L	*Homo sapiens*	H3BQJ8
102	LY6L	Lymphocyte antigen 6L	*Mus musculus*	H3BJG9
103	PATE1	Prostate and testis expressed protein 1	*Homo sapiens*	Q8WXA2
104	PAT13	Prostate and testis expressed protein 13	*Mus musculus*	D3YWX3
105	PAT14	Prostate and testis expressed protein 14	*Rattus norvegicus*	Q9QXN2
106	PAT14	Prostate and testis expressed protein 14	*Mus musculus*	Q3UN54
107	PATE2	Prostate and testis expressed protein 2	*Mus musculus*	Q3UW31
108	PATE2	Prostate and testis expressed protein 2	*Homo sapiens*	Q6UY27

No.	Name	Description	Organism	Access code
109	PATE3	Prostate and testis expressed protein 3	*Homo sapiens*	B3GLJ2
110	PATE3	Prostate and testis expressed protein 3	*Mus musculus*	B3GLJ3
111	PATE4	Prostate and testis expressed protein 4	*Mus musculus*	Q09098
112	PATE4	Prostate and testis expressed protein 4	*Homo sapiens*	P0C8F1
113	PSCA	Prostate stem cell antigen	*Homo sapiens*	O43653
114	PSCA	Prostate stem cell antigen	*Mus musculus*	P57096
115	PIP1	Protein PIP-1	*Sus scrofa*	P83106
116	SLURP2	Secreted Ly-6/uPAR domain-containing protein 2	*Homo sapiens*	P0DP57
117	SLURP2	Secreted Ly-6/uPAR domain-containing protein 2	*Macaca mulatta*	P0DP61
118	SLURP2	Secreted Ly-6/uPAR domain-containing protein 2	*Mus musculus*	P0DP59
119	SLURP1	Secreted Ly-6/uPAR-related protein 1	*Homo sapiens*	P55000
120	SLURP1	Secreted Ly-6/uPAR-related protein 1	*Mus musculus*	Q9Z0K7
121	SOLD1	Secreted protein of Ly-6 domain 1	*Bos taurus*	P83107
122	SACA4	Sperm acrosome membrane-associated protein 4	*Mus musculus*	Q80ZQ0
123	SACA4	Sperm acrosome membrane-associated protein 4	*Bos taurus*	Q32PB3
124	SAMP14	Sperm acrosome membrane-associated protein 4	*Homo sapiens*	Q8TDM5
125	UP1	Urinary protein 1	*Rattus norvegicus*	P81827
126	UP2	Urinary protein 2	*Rattus norvegicus*	P81828
127	UP3	Urinary protein 3	*Rattus norvegicus*	P83121
		**Trematoda**		
128	SMP105220	DIF_5	*Schistosoma mansoni*	B8Y6H3
		**Viruses**		
129	CD59	Surface glycoprotein CD59 homolog	*Saimiriine herpesvirus 2 (strain 11)*	Q00996
		**Insecta**		
130	QVR	Protein quiver	*Drosophila melanogaster*	B5A5T4
131	LYNX1	Protein quiver	*Nilaparvata lugens*	C5IWP3
		**Aves**		
132	LY6E	Lymphocyte antigen 6E	*Gallus gallus*	Q90986
133	PSCA	UPAR/Ly6 domain-containing protein	*Meleagris gallopavo*	G1NJY7
		**Amphibia**		
134	WFP	Freeze-responsive liver protein Li16	*Lithobates sylvaticus*	Q9DG71
135	TFP1	MAC-inhibitory protein	*Xenopus laevis*	A0A3S8RK06

No.	Name	Description	Organism	Access code
136	PMFG	Plethodontid modulating factor isoform G	*Plethodon shermani*	Q0GAX2
137	PROD1	Prod 1	*Notophthalmus viridescens*	A8D0E6
138	PROD1	Prod1	*Batrachuperus londongensis*	A0A0F6QEW0
139	PROD1	Prod1	*Cynops orientalis*	A0A0F6QFB4
140	PROD1 long form	Prod1 long form	*Aneides lugubris*	A0A0F6QDT3
141	TFP2	Tfp2	*Xenopus laevis*	A0A3S8RJZ9
142	TFP3	Tfp3	*Xenopus laevis*	A0A1L8F8M0
143	TFP4	Tfp4	*Xenopus laevis*	A0A3S5HJM2
144	TFP5	Tfp5	*Xenopus laevis*	A0A1L8G0B7
145	TFP6	Tfp6	*Xenopus laevis*	A0A3Q8S4U8
146	LYPD6	Ly6/PLAUR domain-containing protein 6	*Xenopus laevis*	A0A1L8ENX6
**Actinopterygii**				
147	CD59	CD59	*Danio rerio*	A0A0B5JW41
148	LY2.3	Cocaine-and amphetamine-regulated transcript protein-like 1	*Danio rerio*	A0A2R8RTX1
149	LYPD6	Ly6/PLAUR domain-containing protein 6	*Danio rerio*	Q66IA6
150	LY2.2	MAC-inhibitory protein	*Danio rerio*	A0A0G2L893
151	protein bouncer	protein bouncer	*Oryzias latipes*	H2LID1
152	protein bouncer	protein bouncer	*Danio rerio*	P0DPQ9
153	LY2.1	Three-finger protein 5	*Danio rerio*	D2CRG0

Table S2. **List of putative three-finger proteins which were identified in the genomes of starfishes**

No.	Name	Organism	Access code
1	LyAr1 (Lystar5)	*Asterias rubens*	XP_033628517.1
2	LyAr2	*Asterias rubens*	XP_033635706.1
3	LyAr3	*Asterias rubens*	XP_033638913.1
4	LyAr4	*Asterias rubens*	XP_033640057.1
5	LyAr5	*Asterias rubens*	XP_033644501.1
6	LyAp1	*Acanthaster planci*	XP_022094712.1
7	LyAp2	*Acanthaster planci*	XP_022094807.1
8	LyAp3	*Acanthaster planci*	XP_022100632.1
9	LyAp4	*Acanthaster planci*	XP_022109332.1
10	LyAp5	*Acanthaster planci*	XP_022111363.1
11	LyAp6	*Acanthaster planci*	XP_022086630.1

Table S3. Pairwise similarity between some TFPs from starfishes and previously known TFPs. Proteins and organisms are listed in the table

	Lynx2 Homo sapiens	LyAr1 Asterias rubens	LyAr2 Asterias rubens	LyAp1 Acanthaster planci	LyAp2 Acanthaster planci	LyAp5 Acanthaster planci
Lynx2 Homo sapiens	100%	50.5%	55.3%	50.5%	58.8%	55.8%
LyAr1 Asterias rubens	-	100.0%	42.2%	85.6%	46.7%	45.1%
LyAr2 Asterias rubens	-	-	100.0%	40.0%	82.9%	54.8%
LyAp1 Acanthaster planci	-	-	-	100.0%	43.3%	41.8%
LyAp2 Acanthaster planci	-	-	-	-	100.0%	58.3%
LyAp5 Acanthaster planci	-	-	-	-	-	100.0%

Table S4. Pairwise similarity between some TFPs from starfishes and previously known TFPs. Proteins and organisms are listed in the table

	LYPD6 Homo_sapiens	LYPD6B Homo sapiens	LyAp4 Acanthaster planci	LyAr4 Asterias rubens
LYPD6 Homo sapiens	100%	61.0%	65.9%	61.2%
LYPD6B Homo sapiens	-	100%	52.9%	55.3%
LyAp4 Acanthaster planci	-	-	100%	95.3%
LyAr4 Asterias rubens	-	-	-	100%

Table S5. Pairwise similarity between some TFPs from starfishes and previously known TFPs. Proteins and organisms are listed in the table

	LYNX1 N. lugens	Mambalgin 1 D._polylepis	PROD1 N. viridescens	LyAr3 Asterias rubens	LyAp3 Acanthaster planci	LyAp6 Acanthaster planci
LYNX1 Nilaparvata_lugens	100%	29.8%	31.2%	38.4%	44.9%	35.6%
mambalgin1 Dendroaspis polylepis	-	100%	30.3%	39.3%	26.5%	22.1%
PROD1 Notophthalmus viridescens	-	-	100%	25.8%	22.1%	26.6%
LyAr3 Asterias rubens	-	-	-	100%	63.1%	44.3%
LyAp3 Acanthaster planci	-	-	-	-	100%	39.2%
LyAp6 Acanthaster planci	-	-	-	-	-	100%

Table S6. Chemical shifts of the signals of backbone atoms of Lystar5 in H_2O solution, 800MHz, pH 7.0, 37˚C.

		Form I					Form II				
No.	Res.	H^N	N	C^α	C	H^α	H^N	N	C^α	C	H^α
0	MET	-	-	-	-	-					
1	LEU	-	-	52.3	171.6	-					
2	GLN	8.06	121.4	51.5	172.7	5.33					
3	CYS	8.42	118.3	49.0	172.1	4.89	8.33	118.5	48.9	-	4.92
4	PHE	8.97	122.1	56.9	173.3	4.84					
5	THR	8.90	113.1	57.6	169.9	4.78					
6	CYS	8.49	114.6	53.6	169.1	4.98					
7	GLU	9.22	122.1	52.3	171.0	5.15					
8	ALA	9.12	128.1	50.3	173.9	3.67					
9	GLU	8.57	119.1	53.3	174.5	4.63					
10	ASP	8.53	115.9	50.2	173.5	5.02					
11	THR	7.41	111.2	56.7	171.3	-					
12	ASN	-	-	53.3	174.8	-					
13	GLU	8.58	117.6	56.1	174.6	4.07					
14	ASN	7.04	114.9	50.6	172.7	-					
15	CYS	7.70	114.5	54.3	171.4	4.71					
16	ASN	7.21	118.8	49.4	171.3	5.44					
17	ILE	6.38	120.0	58.5	173.5	4.07					
18	LYS	8.58	127.0	53.6	172.7	4.54					
19	GLU	7.92	118.1	52.2	173.4	4.61					
20	ALA	-	-	-	-	-					
21	PRO		-	60.9	172.6	-					
22	VAL	7.70	124.9	62.3	174.5	4.26					
23	LEU	7.58	121.9	52.1	172.6	4.45					
24	LYS	8.61	128.6	52.5	172.5	4.61					
25	THR	8.37	118.9	60.5	171.6	4.47	8.45	119.3	60.5	171.7	4.46
26	CYS	9.27	128.3	50.0	172.8	4.73	9.17	128.1	50.0	172.7	4.77
27	THR	8.45	109.8	59.3	174.0	4.53	8.50	110.0	59.3	174.1	4.52
28	SER	-	-	-	-	-					
29	ARG	-	-	53.2	173.3	-					
30	GLN	7.41	119.1	54.1	171.4	4.10					
31	ASP	8.04	121.2	50.2	174.5	4.62					
32	ARG	8.41	121.5	50.6	171.2	4.98					
33	CYS	8.88	116.1	49.4	171.2	5.46					
34	LEU	9.08	126.1	52.6	174.2	5.33	9.16	126.1	52.4	174.3	5.29
35	THR	9.05	123.0	60.2	169.6	5.07	8.96	122.8	60.3	169.8	5.11
36	GLN	9.80	130.0	51.8	170.8	5.40	9.64	129.2	51.8	170.9	5.36
37	VAL	8.75	125.2	57.9	171.5	5.14	8.83	125.7	57.8	171.4	5.10
38	ILE	8.56	123.9	56.9	171.9	4.40	8.53	124.1	57.4	171.7	4.36
39	TYR	8.34	127.3	54.5	170.8	4.32	8.43	128.3	54.3	170.9	4.37
40	SER	6.36	117.3	53.2	172.2	4.76	6.69	117.7	53.4	-	-
41	THR	8.36	121.0	62.5	173.3	3.98					
42	GLU	8.49	121.3	56.6	174.9	-					
43	ARG	7.46	115.9	53.8	174.7	-					
44	GLY	8.01	107.8	43.5	170.8	3.78/	8.03	108.1	43.5	-	3.78/

		Form I					Form II				
No.	Res.	H^N	N	C^{α}	C	H^{α}	H^N	N	C^{α}	C	H^{α}
						3.98					3.98
45	LYS	7.01	114.8	51.9	172.3	4.72					
46	LEU	8.68	123.7	52.1	173.3	4.65					
47	ARG	8.52	120.9	53.4	171.7	4.64					
48	ILE	9.38	130.0	57.8	171.0	4.98					
49	ASP	8.95	123.5	50.8	171.9	5.29					
50	LYS	8.98	119.6	52.9	174.1	5.60					
51	GLU	8.40	117.3	53.1	172.9	4.83	8.40	118.1	53.0	173.1	4.83
52	CYS	8.56	118.4	55.2	172.0	5.23	8.71	118.4	55.3	171.9	5.20
53	THR	9.43	117.6	58.0	169.6	4.75	9.39	117.3	58.1	169.7	4.71
54	THR	8.71	110.3	56.1	173.3	5.33	8.66	110.0	56.1	173.4	5.33
55	GLU	9.56	123.8	58.7	176.0	-	9.51	123.9	58.7	176.0	-
56	ASP	8.62	117.6	54.5	176.4	4.46	8.64	117.6	54.4	176.4	4.46
57	GLY	8.29	111.6	44.0	174.8	3.87/4.06	8.37	111.9	44.0	174.8	3.85/4.06
58	CYS	8.58	123.3	59.4	175.0	4.20	8.59	123.4	59.4	175.0	4.20
59	THR	8.44	120.5	63.9	174.2	4.00	8.37	120.6	63.9	174.1	4.01
60	ALA	8.53	125.7	52.8	177.3	4.18	8.57	125.7	52.9	177.2	4.20
61	ALA	8.31	120.1	52.6	178.2	4.20	8.34	120.2	52.6	178.2	4.20
62	THR	8.24	116.2	64.7	174.9	3.86					
63	THR	8.49	119.5	63.5	174.3	4.01					
64	GLN	8.48	120.2	56.4	177.0	4.07					
65	LEU	8.34	121.6	55.2	177.0	4.24					
66	GLY	7.94	104.1	43.1	172.3	3.90/4.58					
67	LYS	7.21	117.3	55.0	174.4	4.20					
68	ARG	7.74	116.3	53.9	172.3	4.27					
69	TYR	8.45	116.1	54.3	172.7	4.91					
70	PHE	9.63	120.6	54.3	174.8	4.98					
71	CYS	9.48	125.9	51.5	170.6	-					
72	ASP	6.89	123.1	50.5	172.8	4.62					
73	LYS	8.26	121.9	54.9	173.4	2.61	8.23	121.6	54.9	173.4	2.52
74	SER	8.22	114.4	56.6	172.5	4.43					
75	ARG	7.51	122.9	52.1	172.6	4.66					
76	PRO		-	61.9	173.2	-					
77	ALA	7.33	116.1	48.7	173.5	5.16					
78	TRP	8.60	118.7	52.8	170.7	5.47					
79	GLY	8.00	105.4	44.4	169.9	3.15/5.34					
80	CYS	9.46	123.1	52.6	170.0	5.44	9.45	122.8	52.6	170.0	5.45
81	VAL	9.44	123.9	58.9	172.5	4.96	9.44	123.9	58.9	172.5	
82	GLU	9.34	127.2	52.1	172.2	4.81	9.30	127.3	52.1	172.2	4.79
83	CYS	8.86	120.4	51.4	170.2	5.87	8.88	120.6	51.4	170.2	5.84
84	CYS	9.17	118.6	52.3	171.4	5.26					
85	ASP	8.49	119.0	51.2	173.0	5.00					
86	THR	7.59	111.0	57.4	170.6	4.71			57.3	170.5	
87	ASP	8.09	118.4	53.1	175.1	-	8.11	118.7	53.0	175.1	
88	ARG	9.34	113.3	54.3	173.2	3.07	9.23	113.1	54.3	173.4	

No.	Res.	Form I H^N	N	C^α	C	H^α	Form II H^N	N	C^α	C	H^α
89	CYS	7.58	111.9	54.4	170.4	4.56	7.58	111.9	54.7		4.56
90	ASN	8.60	127.1	50.9	175.6	4.51					

Table S7. Pairwise similarity calculated for TFPs from *A. planci*, identified by BLAST search, and previously identified TFPs from *A. rubens* (Lystar1-4) [3].

	XP_022086630.1 (LyAp6)	XP_022094712.1 (LyAp1)	XP_022094807.1 (LyAp2)	XP_022100632.1 (LyAp3)	XP_022109332.1 (LyAp4)	XP_022111363.1 (LyAp5)	XP_033645811.1 (LYSTAR1)	XP_033638895.1 (LYSTAR2)	XP_033638835.1 (LYSTAR3)	XP_033634394.1 (LYSTAR4)
XP_022086630.1 (LyAp6)	100.0%	33.3%	35.6%	46.4%	33.0%	38.6%	37.4%	54.1%	42.7%	40.2%
XP_022094712.1 (LyAp1)	-	100.0%	45.6%	38.9%	49.5%	44.0%	42.6%	42.3%	46.8%	43.6%
XP_022094807.1 (LyAp2)	-	-	100.0%	44.6%	47.2%	59.5%	48.8%	50.5%	38.9%	50.5%
XP_022100632.1 (LyAp3)	-	-	-	100.0%	42.4%	47.8%	41.8%	53.3%	59.0%	50.5%
XP_022109332.1 (LyAp4)	-	-	-	-	100.0%	47.2%	43.0%	42.6%	38.5%	45.4%
XP_022111363.1 (LyAp5)	-	-	-	-	-	100.0%	46.7%	51.6%	45.6%	50.5%
XP_033645811.1 (LYSTAR1)	-	-	-	-	-	-	100.0%	47.1%	40.7%	44.8%
XP_033638895.1 (LYSTAR2)	-	-	-	-	-	-	-	100.0%	46.7%	45.9%
XP_033638835.1 (LYSTAR3)	-	-	-	-	-	-	-	-	100.0%	45.1%
XP_033634394.1 (LYSTAR4)	-	-	-	-	-	-	-	-	-	100.0%

Table S8. Pairwise similarity calculated for TFPs from *A. rubens*, identified by BLAST search and previously identified TFPs from *A. rubens* (Lystar1-4) [S3].

	XP_033628517.1 (LyAr1)	XP_033635706.1 (LyAr2)	XP_033638913.1 (LyAr3)	XP_033640057.1 (LyAr4)	XP_033644501.1 (LyAr5)	XP_033645811.1 (LYSTAR1)	XP_033638895.1 (LYSTAR2)	XP_033638835.1 (LYSTAR3)	XP_033634394.1 (LYSTAR4)
XP_033628517.1 (LyAr1)	100.0%	45.6%	42.7%	47.3%	45.7%	41.5%	40.2%	42.6%	43.6%
XP_033635706.1 (LyAr2)	-	100.0%	43.0%	51.7%	45.1%	53.6%	49.5%	43.3%	44.4%
XP_033638913.1 (LyAr3)	-	-	100.0%	38.7%	44.1%	47.3%	47.8%	55.8%	53.8%
XP_033640057.1 (LyAr4)	-	-	-	100.0%	41.0%	43.0%	42.6%	41.8%	46.4%
XP_033644501.1 (LyAr5)	-	-	-	-	100.0%	47.0%	47.5%	46.3%	46.5%
XP_033645811.1 (LYSTAR1)	-	-	-	-	-	100.0%	47.1%	40.7%	44.8%
XP_033638895.1 (LYSTAR2)	-	-	-	-	-	-	100.0%	46.7%	45.9%
XP_033638835.1 (LYSTAR3)	-	-	-	-	-	-	-	100.0%	45.1%
XP_033634394.1 (LYSTAR4)	-	-	-	-	-	-	-	-	100.0%

Table S9. Primers used for qPCR experiments.

Gene	Forward primer	Reverse primer	Amplicon length
β-ACTIN	AAC CTT CTT GCA GCT CCT CCG	TCT GAC CCA TAC CCA CCA TCA	199
CHRNA4	TCG TCC TCT ACA ACA AGT GAG	GGT CCA GGA GCC GAA TTT CA	93
CHRNA7	AAT GAC TCG CAA CCA CTC ACC	TCT GTC CAA GAC ATT TGC AGC C	116
ACEE	GGG GAG AAA GCG ATT CCA GAA G	ATC CCT CGC TGA ACT ACA CCA C	230
SYN1	TGC CAA TGG TGG ATT CTC CG	CAG CCC AAT GAC CAA ACT GC	148
SYP	TGC CAA CAA GAC GGA GAG TG	GAA TTC AGC CGA CGA GGA GT	151
DLG4	CTA CCA AGA TGA AGA CAC GCC	TCT GTT CCA TTC ACC TGC AAC	184

References

[1] Teufel, F.; Almagro Armenteros, J.J.; Johansen, A.R.; Gíslason, M.H.; Pihl, S.I.; Tsirigos, K.D.; Winther, O.; Brunak, S.; von Heijne, G.; Nielsen, H. SignalP 6.0 Predicts All Five Types of Signal Peptides Using Protein Language Models. Nat Biotechnol 2022, 40, 1023–1025, doi:10.1038/s41587-021-01156-3.

[2]. Pierleoni, A.; Martelli, P.L.; Casadio, R. PredGPI: A GPI-Anchor Predictor. *BMC Bioinformatics* **2008**, *9*, 392, doi:10.1186/1471-2105-9-392.

[3] Shabelnikov, S.V.; Bobkov, D.E.; Sharlaimova, N.S.; Petukhova, O.A. Injury Affects Coelomic Fluid Proteome of the Common Starfish, Asterias Rubens. *J Exp Biol* **2019**, *222*, jeb198556, doi:10.1242/jeb.198556.
